# Melanocortin receptor accessory proteins in adrenal disease and obesity

**DOI:** 10.3389/fnins.2015.00213

**Published:** 2015-06-10

**Authors:** David S. Jackson, Shwetha Ramachandrappa, Adrian J. Clark, Li F. Chan

**Affiliations:** Centre for Endocrinology, William Harvey Research Institute, Barts and the London School of Medicine and Dentistry, Queen Mary University of LondonLondon, UK

**Keywords:** melanocortin receptors, accessory proteins, adrenal function, obesity, knockout mouse model

## Abstract

Melanocortin receptor accessory proteins (MRAPs) are regulators of the melanocortin receptor family. MRAP is an essential accessory factor for the functional expression of the MC2R/ACTH receptor. The importance of MRAP in adrenal gland physiology is demonstrated by the clinical condition familial glucocorticoid deficiency type 2. The role of its paralog melanocortin-2-receptor accessory protein 2 (MRAP2), which is predominantly expressed in the hypothalamus including the paraventricular nucleus, has recently been linked to mammalian obesity. Whole body deletion and targeted brain specific deletion of the Mrap2 gene result in severe obesity in mice. Interestingly, Mrap2 complete knockout (KO) mice have increased body weight without detectable changes to food intake or energy expenditure. Rare heterozygous variants of MRAP2 have been found in humans with severe, early-onset obesity. *In vitro* data have shown that Mrap2 interaction with the melanocortin-4-receptor (Mc4r) affects receptor signaling. However, the mechanism by which Mrap2 regulates body weight *in vivo* is not fully understood and differences between the phenotypes of Mrap2 and Mc4r KO mice may point toward Mc4r independent mechanisms.

## The melanocortin receptor family

Melanocortin receptors (MCRs) comprise a family of five, class A, G protein-coupled receptors designated MC1R-MC5R with diverse physiological roles. MCRs are found in chordates and are regarded as having evolved from a single ancestral receptor, possibly corresponding most closely to MC4R (Dores, 2013). Signaling by MCRs has primarily been observed as occurring through the stimulatory α unit Gs which activates adenylyl cyclase to produce cAMP, although other pathways have been implicated (Yang, 2011). Receptor properties such as biased agonism (MC4R) and constitutive activity (MC3R and MC4R) have also been reported (Nijenhuis et al., 2001; Breit et al., 2011).

The melanocortin agonist ligands for MCRs, adrenocorticotropic hormone (ACTH) and the melanocyte stimulating hormones αMSH, β MSH, and γMSH, are neuropeptides derived by enzymatic cleavage from proopiomelanocortin (Pritchard et al., 2002). Their relative potencies are set out together with the natural antagonists in Table 1. The natural peptide antagonists comprise agouti and agouti related protein (AgRP) (Cone, 2006). Agouti was first discovered as a high affinity MC1R antagonist, following studies on genetic determinants of yellow coat color and obese phenotype observed in agouti mouse strains (Lu et al., 1994). Agouti-related protein (AgRP) was later identified as a MC3R and MC4R antagonistic peptide almost identical in size and genomic structure to Agouti (Ollmann et al., 1997; Shutter et al., 1997). Since then it has been shown that both Agouti and AgRP can act as inverse agonists in cAMP assays inhibiting MC1R and MC4R constitutive activity respectively (Vage et al., 1997; Chai et al., 2003). However, such inverse agonists to the MC4R can act as agonists through intracellular ERK1/2 signaling (Breit et al., 2011; Mo and Tao, 2013). A number of additional molecules have been shown to alter MCR function in a variety of ways including β-defensin 3 (Beaumont et al., 2012; Swope et al., 2012), attractin (also known as mahogany), and mahogunin (He et al., 2001).

**Table 1:** **Natural ligands of the melanocortin receptor family (Kiefer et al., 1997; MacNeil et al., 2002; Cone, 2006)**.

**Receptor**	**Relative potency of agonists**	**Antagonists**	**Main site of expression**	**Primary Function**
MC1R	αMSH = β MSH = ACTH > γMSH	Agouti	Melanocytes	Pigmentation, inflammation
MC2R	ACTH only		Adrenal cortex	Adrenal steroidogenesis
MC3R	αMSH = β MSH = ACTH ≈ γMSH	Agouti; AgRP	CNS, GI tract, Kidney	Energy homoestasis, inflammation, Entrainment to meal intake
MC4R	αMSH = β MSH = ACTH >> γMSH	Agouti; AgRP	CNS	Energy homeostasis, thermogenesis, appetite regulation, erectile function
MC5R	αMSH > β MSH = ACTH >> γMSH		Exocrine cells	Exocrine function, regulation of sebaceous glands

### MC1R

Pigmentation and the control of the inflammatory functions of the immune system are dependent on MC1R. Stimulation of MC1R in skin and hair follicle cells by αMSH results in melanogenesis producing dark skin or hair in many species, including humans (Beaumont et al., 2011). Variants in MC1R in humans are associated with red hair, pale skin, and increased risk to skin cancer. This action of αMSH is antagonized *in vivo* by agouti (Lu et al., 1994). MC1R is present on human leukocytes, and on murine macrophages, and has been implicated in the anti-inflammatory properties of αMSH (Star et al., 1995; Catania et al., 2004).

### MC2R

MC2R is unique as it is the only MCR that binds to a specific ligand ACTH. The receptor is predominantly expressed in the adrenal gland. In humans, the inability of ACTH to activate MC2R leads to failure of the adrenal gland to generate cortisol, resulting in the potentially lethal condition of familial glucocorticoid deficiency (FGD) (Clark et al., 1993). Mutation in MC2R leads to FGD type 1. The FGD phenotype was reproduced in the MC2R knockout mouse, although the mice also had evidence of mineralocorticoid deficiency (Chida et al., 2007). MC2R is also found in human and mouse bone where it is thought to be involved in osteoblast proliferation (Zhong et al., 2005). Interestingly, patients with FGD type 1 have tall stature (Elias et al., 2000). MC2R has also been detected in human subcutaneous fat (Smith et al., 2003) and the developing testis (O'Shaughnessy et al., 2007). In mouse adipose tissue, MC2R may be implicated in the release of non-esterified fatty acids from adipocytes (Moller et al., 2011).

### MC3R

MC3R is primarily expressed in the central nervous system in the arcuate nucleus of the hypothalamus and limbic areas, where it affects food utilization/partitioning and food anticipatory behavior (Sutton et al., 2008, 2010; Begriche et al., 2011). Although loss-of-function mutations in MC3R have been identified in humans with obesity, there is still uncertainty if these variants are truly causative (Lee et al., 2007; Mencarelli et al., 2011). In mice, MC3R is essential for the maintenance of a circadian rhythm of activity related to feeding behavior (Begriche et al., 2012). Both central and peripheral MC3R are involved in energy utilization (Begriche et al., 2011). MC3R has also been implicated in the anti-inflammatory processes in murine macrophages (Getting et al., 2006; Leoni et al., 2008; Montero-Melendez et al., 2014).

### MC4R

MC4R is thought to bind principally to αMSH in the paraventricular nucleus (PVN) of the hypothalamus (CNS), a region crucial in the control of food intake. Global homozygous deletion of MC4R in mice results in hyperphagia, increased fat and lean mass, increased body length, reduced activity, and reduced metabolic rate (Huszar et al., 1997; Balthasar et al., 2005). Inactivating mutations in MC4R are the single most common form of monogenic obesity in humans (Farooqi et al., 2003). Common variants near the MC4R locus are associated with adiposity, body weight, risk of obesity and insulin resistance at a population level (Chambers et al., 2008; Loos et al., 2008). The function of MC4R has also expanded over recent years and involvement in autonomic regulation of thermogenesis and glycaemia (Berglund et al., 2014), regulation of sympathetic and parasympathetic control of blood pressure (Sohn et al., 2013) and anhedonia (Lim et al., 2012) have all been described. A role in erectile function and sexual behavior have also been reported (van der Ploeg et al., 2002). Most recently, MC4R expression has been demonstrated in enteroendocrine L cells and regulates the release of peptide YY (PYY) and glucagon-like peptide 1 (GLP-1) (Panaro et al., 2014).

### MC5R

The need for MC5R activity in the secretory function of exocrine glands is well-known (Chen et al., 1997; van der Kraan et al., 1998). MC5R is widely expressed and mice deficient of Mc5r have impaired water repulsion and thermoregulation (Chen et al., 1997). There are some suggestions that MC5R expression in the zona glomerulosa of the adrenal gland may be involved in melanocortin stimulated aldosterone secretion (Vinson et al., 1980; Griffon et al., 1994; van der Kraan et al., 1998), although this is not in keeping with the lack of an adrenal phenotype in Mc5r knockout mice (Chen et al., 1997). Stimulation of MC5R in 3T3-L1 adipocytes with αMSH has been shown to result in lipolysis, through cAMP production, and impairment of re-esterification of fatty acids, through the ERK1/2 pathway (Rodrigues et al., 2013).

## The melanocortin receptor accessory proteins, MRAP, and MRAP2

### Discovery of MRAP and MRAP2

The discovery of MRAP (sometimes referred to as MRAP1) in 2005 by Metherell et al. has provided insight into a novel aspect of MCR regulation, previously unknown (Metherell et al., 2005). By studying the clinical condition FGD it was noted that only approximately 25% of FGD cases were attributable to mutations of MC2R (Chan et al., 2009). Failure of MC2R to traffick to the cell surface in cells other than those derived from an adrenal lineage suggested the presence of an adrenal specific accessory protein (Noon et al., 2002). Using whole genome SNP array genotyping on informative families, mutations in the gene encoding a protein derived from open reading frame 61 of human chromosome 21 (C21orf61), corresponding to a murine adipocyte transmembrane protein (Xu et al., 2002), was identified to cause FGD type 2 (Metherell et al., 2005). This gene was subsequently named melanocortin-2-receptor accessory protein (MRAP). The human MRAP gene contains six exons, exons 5 and 6 are alternatively spliced to give rise to two MRAP isoforms, MRAPα (exon1–5), and MRAPβ (exon 1–4 and 6). Exons 1 and 2 are not translated in the human isoforms whilst rodents do not have the corresponding exons 1 and 2 and produce only one form of the protein (Webb and Clark, 2010). The translated small single pass transmembrane domain protein differs from other known GPCR accessory proteins (Webb and Clark, 2010) and has been shown to adopt a unique anti-parrallel homodimer conformation at the cell surface (Sebag and Hinkle, 2007; Cooray et al., 2008). Both isoforms are present with MC2R in human adrenal tissue (Metherell et al., 2005). The presence of either MRAPα or MRAPβ is essential for MC2R cell surface expression and signaling (Metherell et al., 2005; Roy et al., 2007; Cooray et al., 2008; Hinkle and Sebag, 2009), but the response of MC2R to ACTH may differ between the two isoforms, with MRAPα increasing potency but MRAPβ increasing maximal response (Roy et al., 2007).

Human C6orf117 was identified as a possible paralog to MRAP (Metherell et al., 2005). This gene was subsequently designated MRAP2. The human MRAP2 gene has four exons, exons 2–4 code for a 205 amino acid residue protein (Chan et al., 2009). MRAP2 is thought to adopt a similar dual topology at the cell surface (Chan et al., 2009). Mouse Mrap2 gene differs in having two small untranslated exons 1 and 1a (Asai et al., 2013). Zebra fish has a single Mrap and two forms of Mrap2, designated mrap2a and mrap2b (Agulleiro et al., 2010). Mrap2a and mrap2b appear to have different actions and appear at differing time points in zebrafish development (Sebag et al., 2013). Study of the conservation between the Mraps of lampreys, cartilaginous fish, teleosts, and tetrapods has indicated that MRAP2 is the ancestral gene (Webb and Clark, 2010; Dores, 2013).

### Tissue expression of MRAPs

Both MRAPα mRNA and MRAPβ mRNA are found in human adrenal tissue, testis, breast tissue, ovary, fat, skin, and jejunum, MRAPα mRNA alone being more widely distributed in digestive tract tissues, the immune system, and in thyroid and pituitary, and MRAPβ mRNA alone appearing in brain (Metherell et al., 2005). Evidence for human MRAP mRNA has been detected in the hippocampus, prefrontal cortex, cerebellum, and spinal cord, among other tissues (Gardiner et al., 2002). MRAP expression clearly extends beyond MC2R expression, where expression of MC2R has been reported in the adrenal, bone, adipose tissue, ovaries, testes, skin, and the pituitary (Metherell et al., 2005). MRAP2 mRNA was detected in human adrenal and brain tissue (Chan et al., 2009). In mice Mrap2 mRNA was detected by RT-PCR in a wider number of tissues including hypothalamus, pons, brainstem, cerebellum, eye, thymus, pituitary, adrenal, gonads, skin, and fat (Asai et al., 2013). A recent report demonstrated that MRAP2 expression in human endometrium was significantly down regulated during endometrial transition from its pre-receptive state to the receptive state (Hu et al., 2014), although the physiological significance of this change is unknown.

### MRAPs and melanocortin receptors *in vitro*

Several groups have studied the effects of MRAPs on MCR cell surface expression and function in a number of heterologous systems. The absolute requirement of MRAP for the MC2R trafficking and function is clear and recapitulated by many groups (Metherell et al., 2005; Roy et al., 2007; Sebag and Hinkle, 2007). Transient transfection of MRAPα and MRAP2 with MCRs into CHO cells confirmed that MRAP is necessary for surface expression of MC2R and showed that MRAP2 also can enable surface expression of MC2R (Chan et al., 2009). One study using HEK293 cells reported MC2R surface expression in the absence of MRAP (Roy et al., 2007), although this may well have been due to endogenous MRAP2 in some HEK293 cell-lines (Roy et al., 2010). In addition to surface expression, there are suggestions that MRAPs could influence the post-translational glycosylation of MC2R and MC4R (Kay et al., 2013a). MRAP is essential for MC2R to respond to ACTH and although the presence of MRAP2 can enable MC2R to respond to ACTH this is unlikely to be of physiological significance (Gorrigan et al., 2011). The interplay between MRAP and MRAP2 on MC2R function is less clear. Some suggest that MRAP and MRAP2 act in an antagonistic manner whilst others show no effect or an additive effect on MC2R function in the presence of both MRAPs (Chan et al., 2009; Agulleiro et al., 2010; Sebag and Hinkle, 2010).

The reported results on other MCRs vary, which in part may be due to differences in ligands, cell-lines, ratio of MRAP to MCR expressed or be dependent on the ortholog studied. MRAPs have no effect on the trafficking of MC1R and MC3R but reduce surface expression of MC4R and MC5R (Chan et al., 2009; Sebag and Hinkle, 2009, 2010). In the case of MC5R, MRAP appeared to disrupt MC5R dimerization (Sebag and Hinkle, 2009). Reduced cAMP generation in response to NDP-MSH was demonstrated for human MC1-5R (Chan et al., 2009). Other groups have also shown a significant reduction in MC5R signaling in the presence of MRAPs (Sebag and Hinkle, 2010; Kay et al., 2013b). These studies however found no change in MC4R potency (Sebag and Hinkle, 2010) or an increase in MC4R function (Asai et al., 2013; Sebag et al., 2013). MC4R constitutive activity does appear to be affected in the presence of MRAP/MRAP2 (Asai et al., 2013; Kay et al., 2013b; Sebag et al., 2013). The two isoforms of Mrap2 in zebrafish appears to have differing effects on Mc4r function. Mrap2a inhibits activation of Mc4r whilst Mrap2b suppressed the constitutive activity of the receptor but greatly increased the potency of αMSH (Sebag et al., 2013).

## MRAPs and adrenal disease

### MRAP

Loss-of-function mutations in MRAP give rise to FGD type 2 (Metherell et al., 2005). Patients with FGD type 2 present with symptoms and signs resulting from isolated glucocorticoid deficiency and excess plasma ACTH (Chung et al., 2010). FGD type 2 patients present significantly earlier than those with FGD type 1 individuals harboring MC2R mutations, with the exception of those patients with missense MRAP mutations who present later and with a milder phenotype (Hughes et al., 2010).

In keeping with the importance of MRAP's role in glucocorticoid production, with both MRAP and MC2R are highly expressed in the zona fasciculata. However, the highest levels of expression are found in the undifferentiated zone, believed to contain adrenal stem cells, suggesting that MC2R and MRAP maybe important in adrenal development and/or maintenance (Gorrigan et al., 2011). The adrenal histology from deceased FGD patients would support this notion revealing glomerulosa cell disorganization and loss of fasciculata and reticularis cells (Clark and Weber, 1998).

MRAP is a critical component of the hypothalamic-pituitary-adrenal axis, involved in adrenal responsiveness to ACTH and possibly other adrenal disease processes. In rats, the transcription of Mrap RNA closely tracks the normal ultradian pulses of ACTH and, together with similar patterns of transcription and related protein processing of other components of adrenal steroidogenesis, suggests that Mrap protein availability is closely tied to need for signaling in response to ACTH (Liu et al., 2003).

One study assessed MC2R, MRAP, and MRAP2 expression in human adrenal tissue derived from normal and hyperplastic adrenal gland, and from adrenocortical adenomas and carcinomas (Hofland et al., 2012). Their data suggested that the effect of ACTH stimulation on the expression of the ACTH receptor complex comprising MC2R, MRAP and MRAP2 assists in the production of a functioning complex, although the level of MRAP2 being insufficient to reduce its sensitivity to ACTH.

### MRAP2

*In vitro*, MRAP2 has been shown to enable MC2R trafficking to the cell surface and subsequent signaling. N-linked glycosylation appears critical in this process (Chan et al., 2009). The dose of ACTH required to activate the receptor is however 1000 times higher than that compared to MRAP (Sebag and Hinkle, 2010; Gorrigan et al., 2011), which would explain the inability of MRAP2 to rescue MC2R function in patients with MRAP mutations. Furthermore, significantly lower levels of Mrap2 expression compared with Mrap expression are found in adult rat adrenal gland. Unlike Mrap, which is highly expressed in the zona fasciculata, Mrap2 appears sparsely expressed throughout the adult adrenal cortex (Gorrigan et al., 2011). Although, Mrap2 appears highly expressed in the developing adrenal gland, to date no adrenal phenotype have been described in the Mrap2 KO mice (Gorrigan et al., 2011; Asai et al., 2013).

## MRAPs and obesity

### MRAP2

MRAP2 was shown to interact with all MCRs and the expression in the hypothalamus and PVN pointed to a role in central melanocortin regulation of metabolism and appetite (Chan et al., 2009). A recent publication describing obesity in rodents and humans with MRAP2 deficiency has demonstrated that this is indeed the case (Asai et al., 2013). Furthermore, zebrafish deficient of MRAP2 isoforms was shown to have disrupted growth and development supporting the role of MRAP2 in metabolism homeostasis (Sebag et al., 2013).

Phenotypically, global Mrap2 KO mice on an sv129 genetic background fed a chow *ad libitum* diet develop severe obesity at a young age and were found to be significantly heavier than their wild type counterparts at approximately 6 weeks of age. Mrap2^−/−^ mice have increased body length and fat deposits, whilst percentage lean mass was reduced. Heterozygous Mrap2^+/−^ mice have an intermediate phenotype (Asai et al., 2013).

Serum leptin was elevated in Mrap2 null mice, which normalized following weight loss through food restriction. No differences in fasting serum insulin concentration, response to glucose load or serum T3 and T4 levels were detectable between null and wild-type animals, whilst Mrap2^−/−^ males have lower 24 h urine epinephrine and norepinephrine. AgRP mRNA levels in the hypothalamus are increased in Mrap2 null animals without changes in POMC mRNA. Although brown fat deposit was enlarged in obese mice, response to cold challenge normal and Ucp1 mRNA levels in brown fat increased appropriately when mice were subjected to 4°C for 18 h (Asai et al., 2013).

Interestingly, no increase in food intake was detected and despite paired feeding, Mrap2^−/−^ mice became significantly heavier in weight compared with Mrap^+/+^ littermates. Food restriction in Mrap2^−/−^ mice (90% (females) or 87% (males) of wild type intake) was required to normalize their weight gain to that of wild-type Mrap2^+/+^ mice. Faecal energy content was also indistinguishable between Mrap2^−/−^ and Mrap2^+/+^ mice (Asai et al., 2013).

Analysis of young mice prior the divergence of weight between null and wild-type mice demonstrated indistinguishable 24 h energy expenditure and respiratory exchange ratio (RER) when measured by indirect calorimetry. There was no difference in locomotor activity during the day or night and body temperature even when challenged by cold was not different (Asai et al., 2013).

Asai et al. demonstrated that Mrap2 was expressed in several sites of the mouse brain and in the PVN co-localized with Mc4r expressing neurons (Asai et al., 2013), suggestive of an MC4R dependent mechanisms. Mrap2^−/−^ mice share some phenotypic similarities with Mc4r KO mice, which are heavier with increased length and adiposity. Heterozygous Mc4r^+/−^ mice have an intermediate weight phenotype (Huszar et al., 1997). In support of an MC4R mechanism, mice with conditional deletion of Mrap2 in Sim1 neurons that express Mc4r were equally obese and phenotypically similar to global Mrap2^−/−^ mice (Asai et al., 2013). However, differences exist between Mrap2^−/−^ and Mc4r^−/−^ mice. In Mc4r^−/−^ mice, obesity is attributable to hyperphagia and reduced energy expenditure. The Mc4r null mice also have increased lean mass (Balthasar et al., 2005; Sutton et al., 2006).

In humans, disabling mutations of MC4R are the most common cause of monogenic obesity and found in up to 6% of severely obese patients (Vaisse et al., 1998; Yeo et al., 1998). In comparison, genetic screening of two large obese pediatric cohorts identified only four rare heterozygous MRAP2 variants (N88Y, L115V, R125C, E24X) in patients with severe early onset obesity (Asai et al., 2013). The individual carrying the most damaging variant, E24X variant, was the most severely affected with a reported BMI of 63 kg/m2 (BMI SDS 4.7) at the age of 19 years.

Data from zebrafish supports the notion that MRAP2 is an MC4R accessory protein capable of regulating the function of MC4R (Asai et al., 2013; Sebag et al., 2013). However, several lines of evidence from the Mrap2^−/−^ mice data would suggest the possibility of Mc4r independent mechanisms. Firstly, Mrap2^−/−^ mice remain responsive to treatment with MTII, a Mc3r and Mc4r agonist, whilst anorexic response to MTII is abolished in Mc4r^−/−^ mice. Secondly, Mrap2^−/−^ and Mc4r^−/−^ double KOs are less obese compared with Mc4r^−/−^ mice alone and finally the Mrap2^−/−^ does not completely replicate Mc4r^−/−^ mice phenotype.

The phenotype of the Mc3r homozygous knockout mice is of interest. Mc3r^−/−^ animals are not obviously hyperphagic but eventually become heavier than wild type mice on a standard chow diet, and have more adipose tissue and smaller bones, but no significant difference in energy expenditure (Chen et al., 2000; Begriche et al., 2011). Heterozygous Mc3r knockout mice are similar to wild type mice (Chen et al., 2000). More recently it has been shown that MRAP2, but not MRAP, localizes at the apical plasma membrane in the presence of MC3R in a polarized cell, MC3R also localizing at the apical membrane (Park et al., 2014). Receptors and other structures in neurons have precisely determined locations and are associated with dedicated trafficking mechanisms in which MRAPs may have a role.

There is no suggestion that Mrap2 plays a role in MC1R or MC2R function as Mrap2^−/−^ mice have normal coat color and corticosterone production (at baseline and when stressed) (Asai et al., 2013).

### MRAP

It is not yet known if MRAP is associated with mammalian obesity. There is a single case report describing a family with a splice site mutation of MRAP. Family members homozygous and heterozygous for the mutation were obese compared with normal weighted unaffected members (Rumie et al., 2007). As yet the knockout mouse has not been reported. In murine 3T3-L1 cells, ACTH triggers lipolysis and knockdown of Mrap in these cells substantially inhibits lipolysis (Kim et al., 2013). Furthermore, the promoter of Mrap was found to include a region binding the transcription factor PPARγ that regulates adipogenesis in fibroblasts (Tontonoz et al., 1994; Kim et al., 2013).

## Future of MRAPs

**Figure 1:**
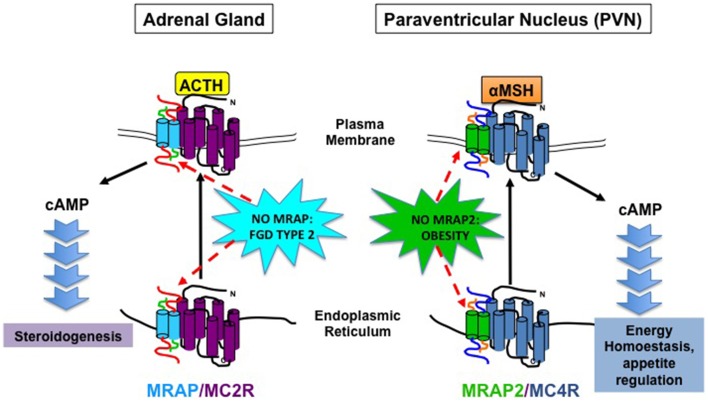
**Schematic Diagram illustrating MRAP and MRAP2 action on MC2R and MC4R, respectively and physiological consequence of MRAP and MRAP2 deficiency on adrenal steroidogenesis and energy homeostasis**.

The discovery of MRAP and MRAP2 has initiated a paradigm shift in our understanding of MCR and GPCR signaling (Figure 1). For MRAP, the ability to functionally express MC2R in non-adrenal cell lines has opened up many opportunities including screening for MC2R peptide antagonists (Bouw et al., 2014). Moreover, the finding that MRAP2 is associated with mammalian obesity is exciting and could provide a novel therapeutic target at a time when obesity is at epidemic levels. There are many scientific questions yet to be answered. For example, the precise mechanism of how MRAP2 causes obesity is not fully understood. It is intriguing that no difference in food intake or energy expenditure was detected in Mrap2^−/−^ compared to wild-type littermates. This may represent the lack of sensitivity of the systems in place to detect the relatively small changes in food intake and energy expenditure. If so this demonstrates the fine balance in energy homeostasis, where small changes could tip the scale leading to significant weight gain. However, in light of the differences between Mc4r^−/−^ and Mrap2^−/−^ mice, it is likely that MC4R independent mechanisms as well as MCR independent pathways are at play. The complexity of these proteins and how they regulate MCR and GPCR function is only just beginning to be explored.

### Conflict of interest statement

The authors declare that the research was conducted in the absence of any commercial or financial relationships that could be construed as a potential conflict of interest.
